# Study on Surface Properties of Aramid Fiber Modified in Supercritical Carbon Dioxide by Glycidyl-POSS

**DOI:** 10.3390/polym11040700

**Published:** 2019-04-17

**Authors:** Yang Li, Zhu Luo, Le Yang, Xiaolong Li, Kun Xiang

**Affiliations:** 1 Department of Polymer Material and Engineering, College of Materials and Metallurgy, Guizhou University, Guiyang 550025, China; 20130214@git.edu.cn (Y.L.); leiyang15@gzu.edu.cn (L.Y.); withoutname635@163.com (X.L.); 2 School of Materials Science and Metallurgy Engineering, Guizhou Institute of Technology, Guiyang 550003, China; 3 School of Physics and Electronic Science, Guizhou Education University, Guiyang 550018, China; gzsfxyxk@163.com

**Keywords:** aramid fiber, glycidyl-POSS, supercritical CO_2_ processing, graft reaction, interfacial strength

## Abstract

The outstanding diffusivity and permeability of supercritical carbon dioxide (scCO_2_) are extremely beneficial for grafting reaction. In this work, aramid fibers (AF) are modified in scCO_2_ by glycidyl-polyhedral oliomeric silsesquioxane (POSS) with 2-ethyl-4-methylimidazole (2E4MZ) on the basis of cleaning with acetone. The surface morphology and chemical structure of the modified AF were measured and characterized by Fourier transform infrared spectroscopy (FTIR), X-ray photoelectron spectroscopy (XPS), Scanning electron microscope (SEM), Thermogravimetric (TG), and Atomic force microscope (AFM). The interfacial shear strength (IFSS) was measured by a micro-bond pull-out test, then the modified AF/EP composites were prepared and the interlaminar shear strength (ILSS) was characterized. Research has shown that some of the glycidyl-POSS molecular chains permeated into the surface of the fiber and grafted onto the surface of the AF after modification, and the other glycidyl-POSS self-assembled on the surface of the fiber. XPS indicated the introduction of C–O and –COO–, which confirmed the existence of chemical reactions between AF and glycidyl-POSS. AFM and SEM images revealed that 2E4MZ, not only promoted the grafting reaction of glycidyl-POSS, but also intensified the self-assembly of glycidyl-POSS, both of which increased the roughness of the fiber. A monofilament tensile test and micro-bond pull-out test showed that there was a negative effect on the tensile strength after scCO_2_ processing. The tensile strength of modified AF, with glycidyl-POSS, increased the highest strength of 25.7 cN dtex^−1^, which was 8% higher than that of pristine AF. The improvement of ILS roughness and the polar chemical groups produced in grafting reaction. These results indicated that AF, treated in scCO_2_, with glycidyl-POSS, which is a suitable way of fiber modification, can significantly improve the surface adhesion of AF reinforced composites.

## 1. Introduction

Aramid fiber (AF), also called aromatic polyamide fiber, is a new type of high performance industrial fiber with high modulus, high strength, low density, high temperature resistance, low elongation at a break and low lag loss. However, AF has disadvantages, such as surface smoothness, high crystallinity, and low chemical activity, which result in weak interfacial adhesion between the fiber and matrix materials [1,2,3]. Therefore, the development of novel and effective modification technology, to improve interfacial adhesion, is an issue. In order to improve the surface adhesion between fibers and matrix materials, two ways were used for AF modification. One was a physical method [4,5], and the other was chemical modification. The commonly used chemical modification methods include surface grafting [6,7], surface modification by plasma [8,9], and ultrasonic infiltration [10]. The surface coating as a physical method has been widely used because of simple equipment and convenient operation.

Glycidyl-polyhedral oliomeric silsesquioxane (POSS) is one such hybrid material that possesses both organic and inorganic properties. The chemical structure of these organic groups of glycidyl-POSS can interact with amino and carboxyl [11]. For non-reactive inorganic groups, glycidyl-POSS can show compatibility between the POSS molecule and matrix materials. Because of the high crystallinity on the surface layer of AF, it is difficult for glycidyl-POSS to penetrate into the surface layer of the fiber. Supercritical carbon dioxide (scCO_2_) not only has great solubility and permeability, but also has a relatively high diffusion coefficient, which caused the polymers and fibers to bulge [8,12]. The study on AF modification in scCO_2_ processing by an interfacial reaction between glycidyl-POSS and the polar groups on AF surface has not yet been reported. Traditional surface coating treatment makes it difficult for surface treatment agent to penetrate the surface of aramid fiber. Moreover, stress concentration is easily caused by the uneven grafting point in the process of chemical grafting, which reduces the tensile strength of single fibers [13]. Therefore, it is necessary to find an auxiliary process in the coating treatment and chemical grafting treatment.

Various studies have been carried out in characterizing POSS reinforced materials. These have included studying the formation of nanoscale structures [7,11], the synthesis of novel POSS molecules with different functionalities [14,15], and the enhancement of physical properties [12,16,17]. However, limited work has been reported on the effects of glycidyl-POSS (catalyst is 2-ethyl-4-methylimidazole, 2E4MZ) on grafting modification of AF and reinforcements for the mechanical properties of epoxy resin matrix. Therefore, the objective of this paper is to investigate the modification effect of glycidyl-POSS on AF in scCO_2_ and study the effects on mechanical properties of epoxy resin reinforced by AF.

## 2. Surface Treatment Process and Characterization

### 2.1. Materials

The polymer system, used in this work, consists of glycidyl-POSS (EP0409, PassKey Instrument Co. Ltd, Changsha, China) and 2-ethyl-4-methylimidazole (2E4MZ, Shikoku Chemical Corporation, Shikoku, Japan). Aramid fiber (AF-1000, 1500D) was produced by South Alkex Company, Seoul, Korea. Epoxy resin (EP), E44, ShangHai Resin Factory Co., LTD (Shanghai, China).

### 2.2. Preparation of Modified Aramid Fiber

**Cleaning process of aramid fiber**: Aramid fibers (AF-1000, 1500D; Seoul, Korea) used in this study was produced by South Alkex Company with 0–0.3% surface sizing agent, as stated by manufacturer. The sizing agent on the surface of AF is used to make the fibers antistatic to help with processing and handling. In order to eliminate the effect of sizing agent on grafting reaction, all samples were washed with acetone and rinsed with ethanol and dried in vacuum before surface treatment. 

**Figure 1:**
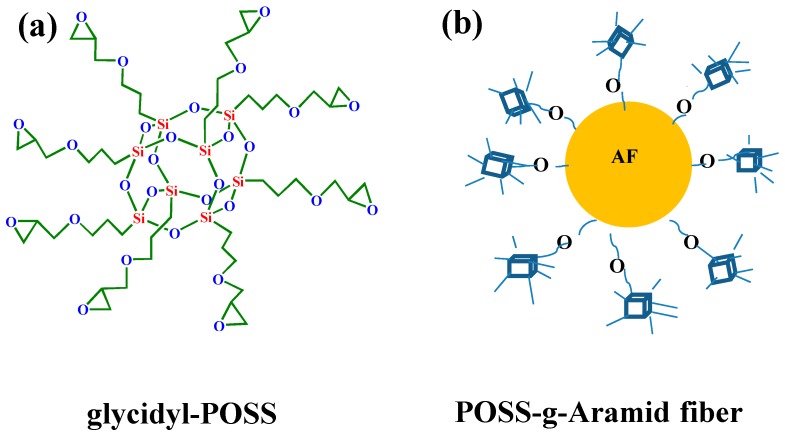
(**a**) Chemical structure of glycidyl-POSS. (**b**) Schematic drawing of glycidyl-POSS-AF.

**Figure 2:**
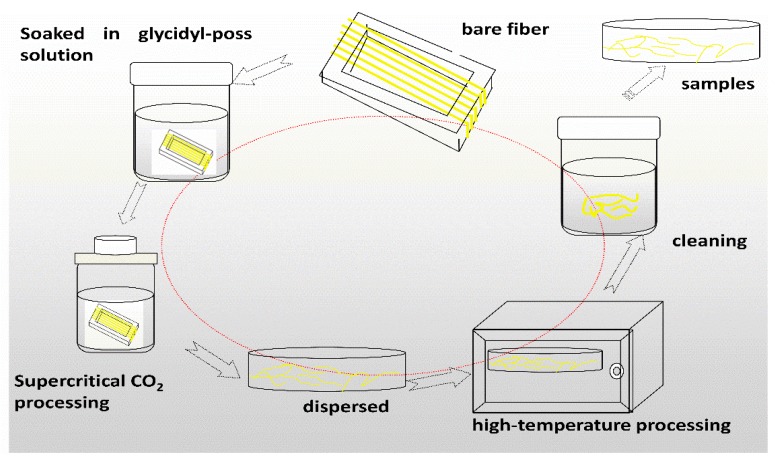
Diagram of glycidyl-polyhedral oliomeric silsesquioxane (POSS) grafting on aramid fibers (AF) preparation process.

**Figure 3:**
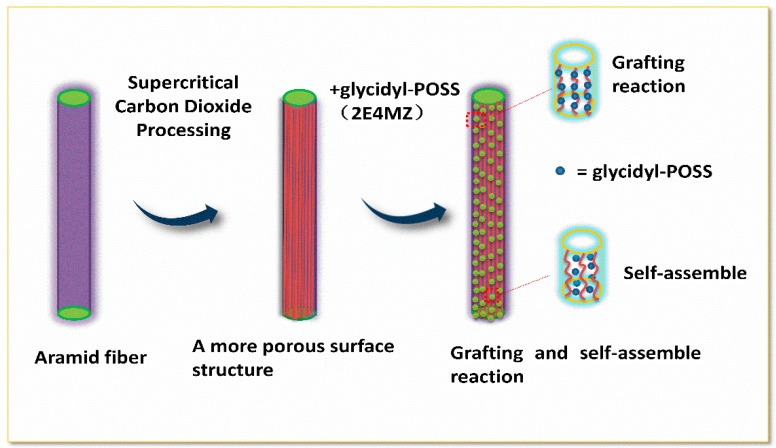
Scheme of the modification process.

**Preparation of samples:** The AF was uniformly mixed with 5 wt % glycidyl-POSS (Chemical structure of glycidyl-POSS is shown in Figure 1a solution (the dosage of 2E4MZ was 5wt % of glycidyl-POSS mass), ultrasonic processing was used in a beaker at 25 °C for 10 min, then scCO_2_ processing was operated (10 MPa, 50 °C, 30 min), left to dry in an oven at 150 °C for 1 h. A large number of fibers may stick together during the processing. The fiber bundles were overturned and scattered before the heat treatment process to prevent fibers forming bundles. The schematic drawing of glycidyl-POSS-AF is shown in Figure 1b. After the graft processing, the specimens were removed from the container, rinsed with acetone to remove the unreacted glycidyl-POSS on the fiber surface and then dried. The schematic diagram of sample processing and grafting diagram are respectively shown in Figure 2 and Figure 3, the samples obtained by different treatment methods are shown in Table 1.

### 2.3. Characterizations

The surface chemical modification of the modified AF was studied using FT-IR (Nicolet 8700, Thermo Fisher Scientific, Waltham, MA, USA), which provided the information about various chemical bonds. TGA was performed on the Thermogravimetric Analyzer (TGA, Q50, DuPont, Delaware, MD, USA) in nitrogen at a ramp rate of 10 °C/min. The samples were analyzed in the range of 80–800 °C. XRD analyses were performed by using a diffractometer with the type of X Pert PRO from Panalytical, Netherlands. Test conditions are following: Cu-Ka radiation, tube voltage with 40 KV, tube current with 40 mA. Scan range from 10 to 40, scan rate with 2/min. In addition, the XPS tests were conducted by X-ray photoelectron spectroscopy (Thermo fisher Scientific, K-Alpha^+^, Waltham, MA, USA). The microstructure of the samples was studied by using field emission scanning electron microscopy (Sirion 200, FEI, Hillsboro, OR, USA). Atomic force microscopy (Bruker, Dimension, Billerica, MA, USA) was used to examine the fiber surface in more detail.

### 2.4. Measurements of the Interfacial Shear Strength (IFSS)

**Figure 4:**
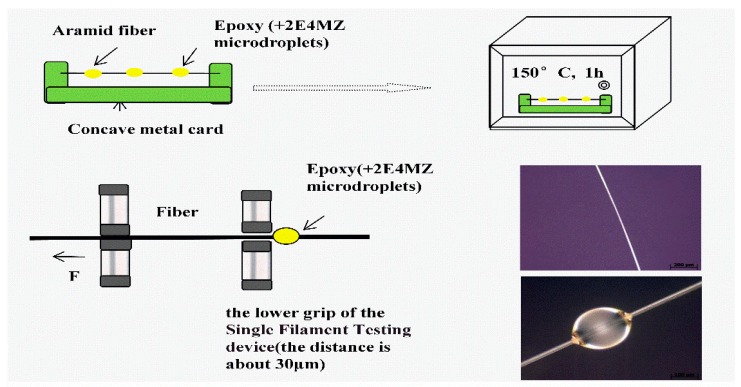
Micro-droplet test.

In order to investigate how processing affects the interfacial shear strength, specimens were made using AF and a certain amounts of epoxy resin E44 (5 wt % 2E4MZ as a curing agent). The micro-bond pull-out test was carried out on a Single Filament Testing device XQ-1 (Shanghai New Fiber Instrument Co. LTD., Shanghai, China) [12]. After specimens were mounted (shown in Figure 4), the bottom fixture was adjusted to make it grip the micro-droplet resin at the bottom. The upper clamping device clamped the fiber and then a steady displacement was applied to pull out of the single fiber from the epoxy droplets with a displacement rate of 10 mm/min. The diameter (**D**) and embedding length (**L**) of the fibers were measured by Inverted Metallographic Microscope (Axio Observer Z1m, Zeiss, Germany). The test was conducted at room temperature, and the maximum load causing debonding was measured. For each group, 20 samples were tested and averaged. Assuming the IFSS is approximately constant along the entire interface, then the average IFSS can be calculated from the following Equation (1):

(1) $$\tau =\frac{F_{d}}{\pi \mathrm{DL}}$$

where τ is the average IFSS, F_d_ is the maximum load of interfacial failure, D is the fiber diameter, and L is the embedded length.

### 2.5. Measurements of the Interlaminar Shear Strength (ILSS)

To investigate how the treatment affects the interlaminar shear strength (ILSS), the ILSS of AF reinforced epoxy composites before and after modification was tested according to ASTM D2344 by using a universal tensile machine (5882, NKKN). The samples were made using AF (10 wt % ± 0.2) and a certain amount of epoxy resin E44 (4 wt % 2E4MZ as a curing agent). The specimens were prepared by moulding, and the pressure and temperature are 10 MPa, and 100 °C, respectively, the pressure holding time is 1 h. For each group, average value was determined from five different samples. The ILSS were calculated by Equation (2):

(2) $$\mathrm{ILSS}=0.75\times \frac{P_{m}}{b\times h}$$

where P_m_ (N) is the maximum load during the test, **b** (mm) is the measured specimen width, and h (mm) is the measured specimen thickness.

## 3. Results and Discussion

### 3.1. FTIR and XPS

**Figure 5:**
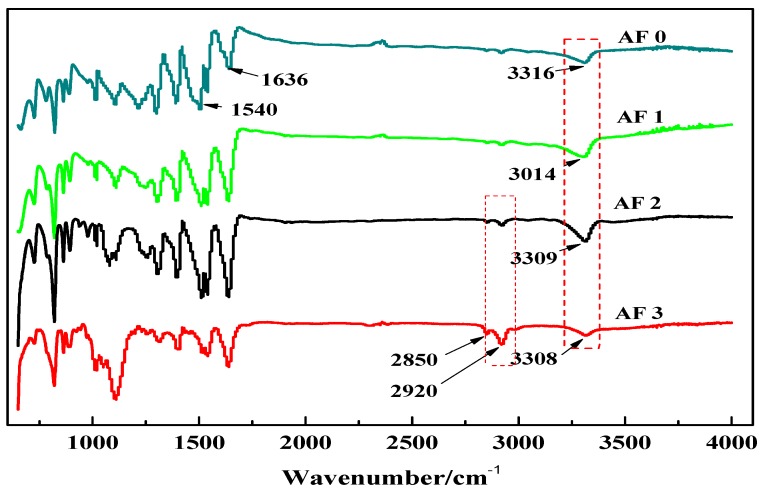
FT-IR spectra of aramid fiber before and after treatment.

**Figure 6:**
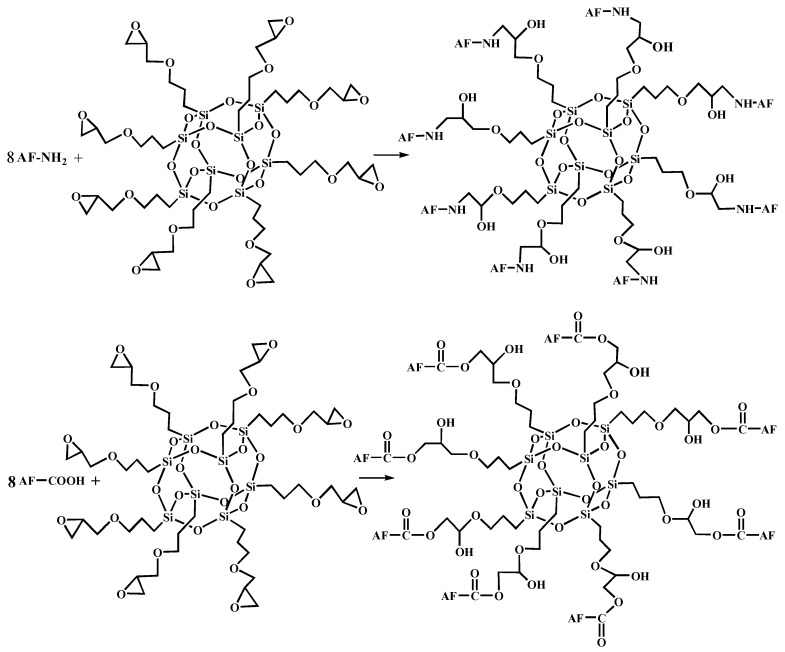
The terminal group reaction between aramid fiber and glycidyl-POSS.

**Figure 7:**
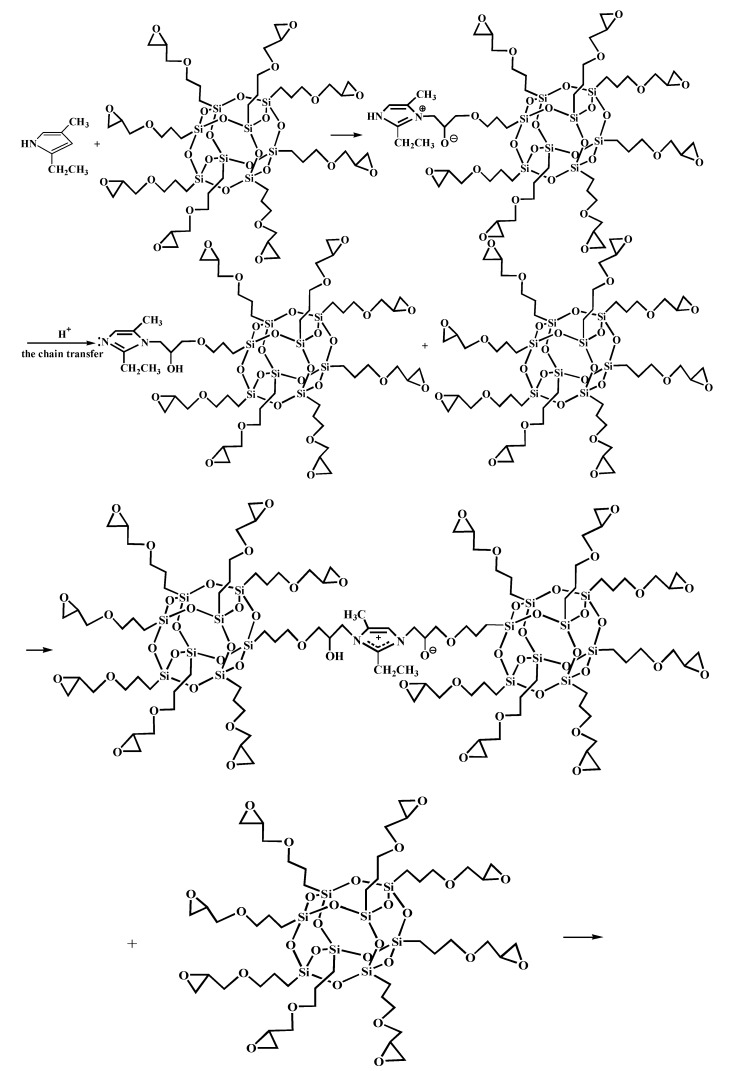

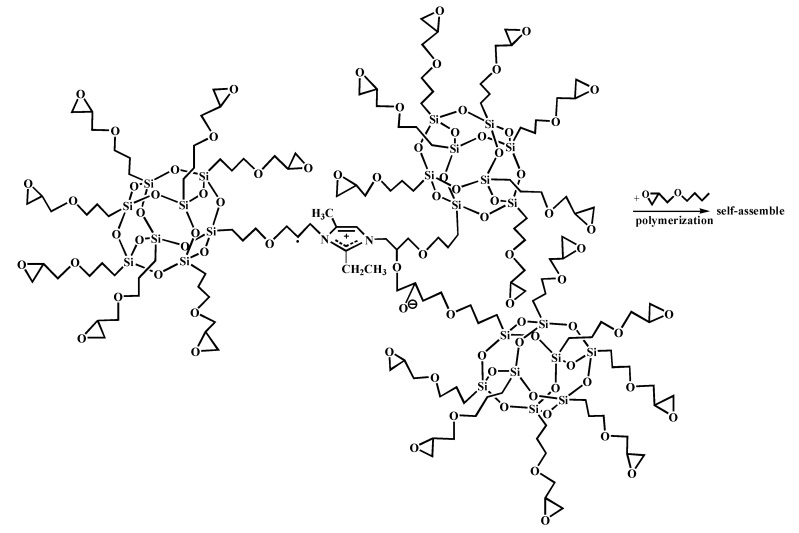
Self-assembly of glycidyl-POSS.

Figure 5 shows the FT-IR spectra of AF before and after modification. The peaks of the AF untreated (AF0) appeared at 3316 cm^−1^ (–NH, derived from hydrogen bond association state), 1636 cm^−1^ (stretching vibration of –C=O, Amide I band), 1540 cm^−1^ (curved vibration of –N–H), 1307 cm^−1^ (bending vibration of –N–H) [17,18,19]. The peak of hydrogen bonds nearby 3310 cm^−1^ was broader and moved to a lower wave-number, which made it clear that the hydrogen bond between the surface molecular chains was weaker after cleaning and coating during scCO_2_ processing. These indicated that scCO_2_ can destroy the surface structure of AF to some extent [11,12]. The peaks around 2900 cm^−1^, which derive from –CH– and–CH_2_– indicated a good dispersion of glycidyl-POSS on the AF surface. In addition, the Si–O–Si stretching peak may be masked by AF peaks between 1095 cm^−1^ and 1118 cm^−1^, which exhibited that glycidyl-POSS was well-consumed in the surface of AF after scCO_2_ processing. Figure 6 and Figure 7 show the mechanism of grafting reaction. Under the action of scCO_2_, the amino and carboxyl groups on the surface of aramid fiber were released. Carboxyl and amino groups attacked the oxygen and carbon atoms of epoxy groups respectively, so that glycidyl-POSS were opened and grafted (Figure 6). Firstly, for 2-ethyl-4-methylimidazole, the third nitrogen atom on the imidazole ring opened the epoxy group, the hydrogen atoms which connect with para-nitrogen atoms caused hydrogen proton transfer and then reacted with the epoxy group to form a 1:2 addition production. Then, the oxygen anion, generated by the epoxy ring opening, continued to catalyze the ring opening polymerization of the epoxy group. This effect does not only promote the self-polymerization of POSS, but also improves the reaction rate of epoxy group with amino and carboxyl groups. (Figure 7).

**Figure 8:**
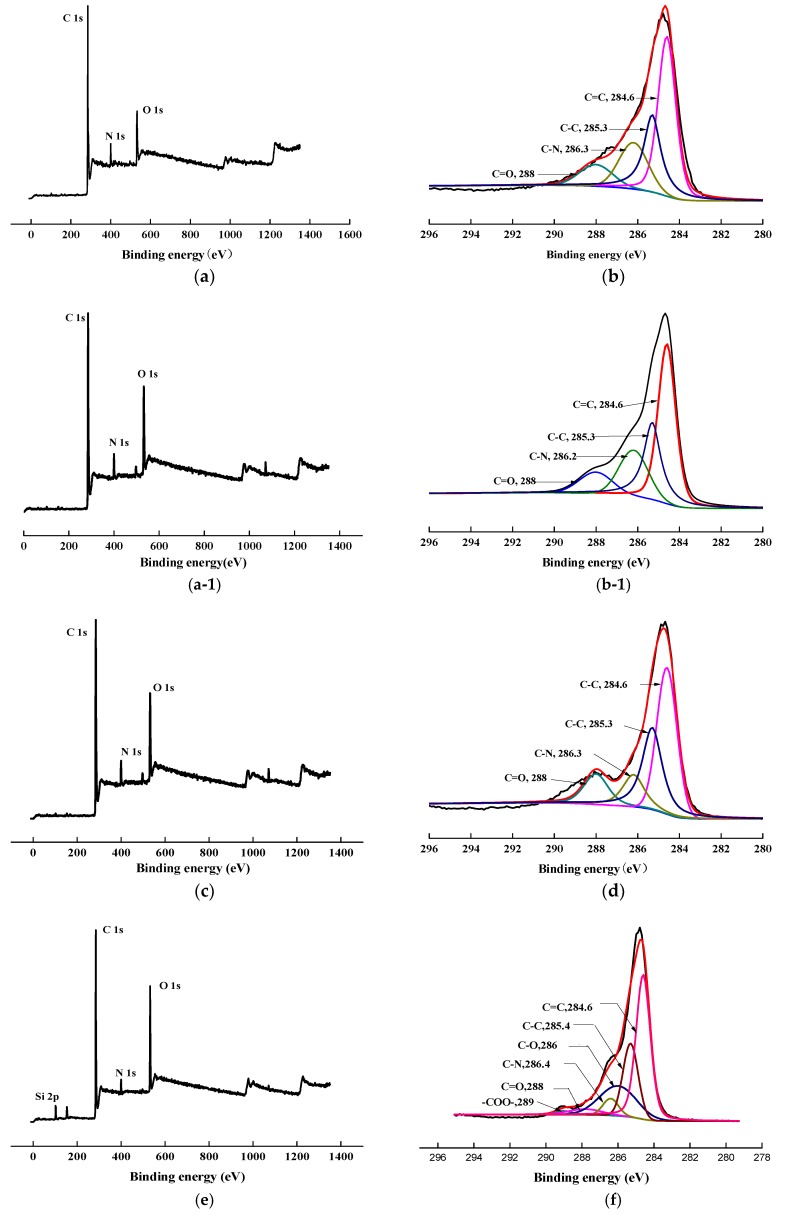

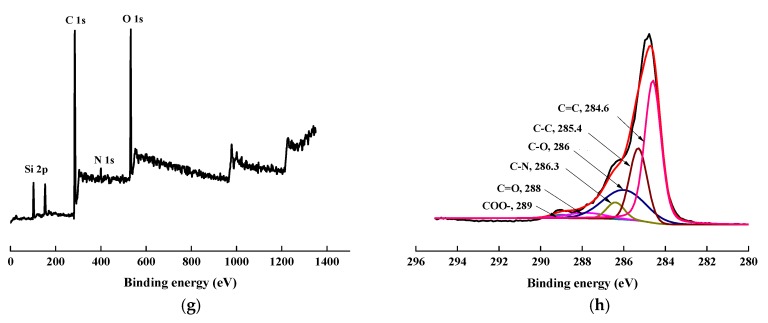
XPS wide scan and C 1s core-level spectra of AF before and after modification. (**a**), (**b**): AF0; (**a-1**), (**b-1**): AF0-T; (**c**), (**d**): AF1; (**e**), (**f**): AF2; (**g**), (**h**): AF3.

To confirm the success of surface modification, XPS tests were conducted. The changes in the chemical structure of the specimens, induced by the scCO_2_, can be observed by analyzing the XPS spectra. The wide scan and C1s core-level spectra of AF before, and after, modification represented in Figure 8, and the results of the analysis were shown in Table 2 and Table 3. As shown in Figure 8a,c,e,g, the wide scan spectrum of AF before, and after modification, showed the same peak components of C 1s, N 1s, and O 1s ascribed to the existence of C, N, and O elements on the surface. As shown in Figure 8(b-1), compared with the theoretical value of N/O for untreated AF (AF0), the oxygen content increased after high temperature processing (O/N = 1.34, AF0 but treated at 150 °C for 1 h), which is consistent with the reported in literature. [20] The content of oxygen increased after scCO_2_ processing (O/N = 1.20, Table 2). The O/C value of CO_2_ was much higher than in AF0, indicating that after scCO_2_ processing, CO_2_ infiltrated into the surface molecular chain of AF in some way [11,12]. The oxygen content of AF2 increased after coating treatment (O/N = 2.54), which proves that glycidyl-POSS has been successfully coated onto the surface of AF. In the same conditions, the oxygen content of AF3 (O/N = 6.08) further increased after using 2E4MZ, which proved that a mass of glycidyl-POSS has been successfully coated onto the surface of AF.

Presented in Figure 8b,d, the C1s core-level spectrum of AF can be curve-fitted with four peak components, whose binding energies located at 288 eV for C=O species, 284.6 eV for C=C species, 286.3 eV for C–N species and 285.3 eV for C–C species [3,9]. In the C1s spectra of AF2 and AF3 there were two more species at the binding energy of about 286 eV and 289 eV assigning to C–O and –COO–, as reported in the literature [14,21]. From the perspective of molecular structure, COO– is only derived from the reaction of glycidyl-POSS with terminal carboxyl groups and the SCCO_2_ processing. C–O not only comes from POSS molecules, but is also derived from the reaction of glycidyl-POSS with terminal amino groups. By further comparing the C1s core-level spectrum of each specimen Figure 8b,d,f,h, a significant change occurred at 286–288 eV. This suggested that the infiltration of the scCO_2_, on the fiber surface, is not only a physical permeation but may also be accompanied by a chemical reaction [12]. Meanwhile, the data in Table 3 indicated that, after using 2E4MZ, the content of C–O increased to 21.74%, but the change of –COO– was not obvious. This phenomenon further confirms that the grafting reaction and self-assembly of glycidyl-POSS occur simultaneously.

### 3.2. XRD Results

**Figure 9:**
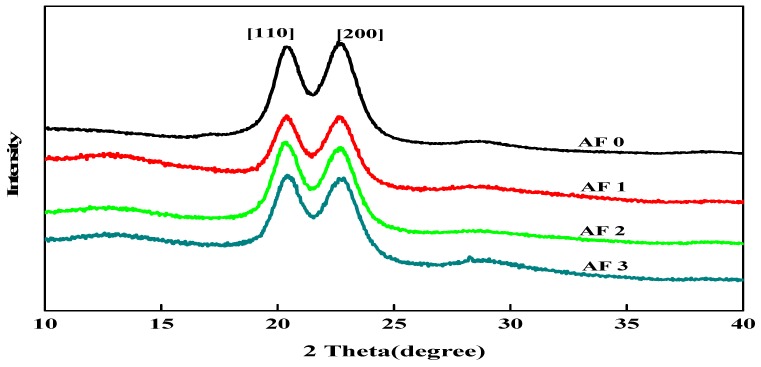
XRD patterns of AF before and after modification.

To clarify the effect of grafting treatment on the surface structure of fibers, XRD measurements were carried out and the X-ray diffractograms in the 2θ range 10° to 40° are shown in Figure 9. It was found that two distinct characteristic diffraction peaks appeared nearby 2θ = 20.0°, 23.8°, which correspond to [110] and [200], indicating that the crystal type of AF does not change after surface cleaning and surface grafting treatment with glycidyl-POSS solution [9,12].

In order to obtain the effect of the treatment process on the structure and crystallinity of AF, the XRD pattern of every specimen was determined in Table 4 by using the curve fitting and normalization method. Table 4 showed the crystal angle value corresponding to the location of each characteristic diffraction peak of AF. After the scCO_2_ processing, the 2θ value slightly shifted to a lower angle in Table 4, indicating that the interplanar spacing of modified AF had increased, and the stacked density of microcrystals had decreased after scCO_2_ processing [12]. After scCO_2_ processing, the crystallinity of the AF1 were 77.10% with a significant drop, which was associated with the destruction of the surface molecular chain. Whether coated or being grafted, the degree of disorder of molecular chains on the fiber surface would increase. Compared with the untreated AF0, the crystallinity of AF3 reduced to 74.54%, which was related to the coated glycidyl-POSS on the surface of fiber.

### 3.3. TG and DTG

**Figure 10:**
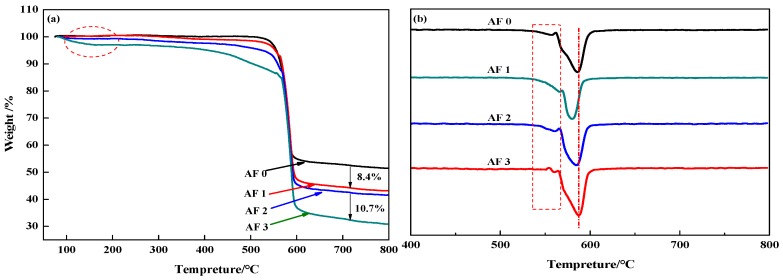
(**a**) TG and (**b**) DTG curves of aramid fiber before and after modification.

Figure 10 showed the TG and DTG curves of aramid fiber before, and after, modification. Although TG curves in Figure 10a showed that there are no distinct difference in AF0 and AF1, AF2 and AF3 had a pyrolysis characteristic in a lower temperature (nearby 150 °C). Meanwhile, both AF1 and AF2 showed an obvious second-order thermogravimetric mechanism, but AF3 showed only a first-order thermogravimetric mechanism and the thermal properties of the fibers improved after glycidyl-POSS (+2E4MZ) processing. These phenomena can be explained as follows [12]: After scCO_2_ processing, the surface structure of the fibers was loosened, the degradation mechanism changed, and the thermal properties decreased. The looser surface structures were favorable for the permeation of glycidyl-POSS, this penetration contributed to the grafting and coating of POSS.

In detail, the pyrolysis characteristic near 150 °C can be attributed to the evaporation of absorbed water and remained glycidyl-POSS (or 2E4MZ). There was almost no weight change within 100–450 °C for AF0 and AF1. According to the analysis of the mass residual rate, the mass residual rate of AF1 is 8.4% lower than in AF0, which may be the residual organic solvent in the processing. By comparing AF1 and AF2, we can draw a clear conclusion that, whether the attachment is a graft product or self-polymerization product, only a little of glycidyl-POSS can attach onto the fiber surface after scCO_2_ processing. Using 2E4MZ can promote the grafting and coating of glycidyl-POSS (AF3). 10.7 wt % residues were the glycidyl-POSS which grafted or coated on the surface of the fibers.

### 3.4. Surface Morphology of the Aramid Fibers

**Figure 11:**
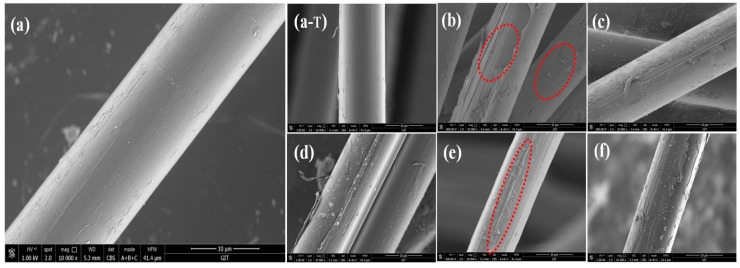
SEM images of aramid fiber before and after modification. ((**a**) AF0; (**b**) AF1; (**c**) AF2 without scCO_2_ processing; (**d**) AF2; (**e**) AF3 without scCO_2_ processing; (**f**) AF3; (**a**-**T**): AF0 sample were treated under 150 °C for 1 h).

The surface morphologies of the specimens were observed by SEM, just as shown in Figure 11 the AF0 displayed a smooth surface, and there was almost no obvious concave and convex structure. As shown in Figure 11(a-T), after high temperature treatment (AF0 but treated at 150 °C for 1 h), there is almost no change on the surface of the fibers. Nevertheless, a significant change has taken place on AF1 after scCO_2_ processing, even part of the surface structure of the fiber was obviously broken. For comparison, the SEM images of AF2 and AF3, which were without scCO_2_ processing, are also listed in Figure 11c,e. It can be clearly seen that a small amount of glycidyl-POSS adhered onto the surface before scCO_2_ processing Figure 11e. After using 2E4MZ, part of glycidyl-POSS penetrated into the fiber through the looser surface, while self-assembly and grafting reactions of glycidyl-POSS occurred on the surface and adhered onto the surface of the fiber. The surface of the fiber was densely coated with a layer of glycidyl-POSS, and the roughness of fibers changed.

### 3.5. Atomic Force Microscopy

**Figure 12:**
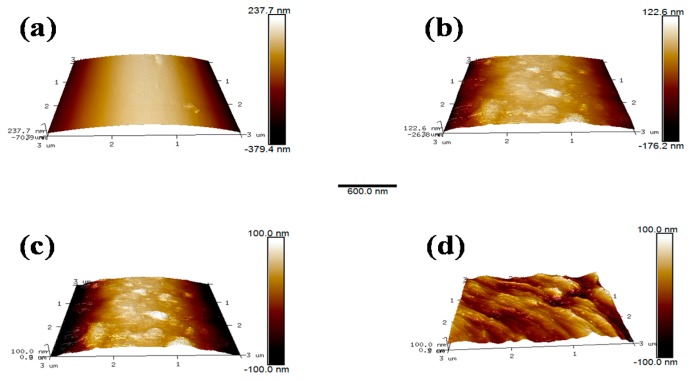
AFM image (3 × 3 μm^2^) of AF before and after modification. ((**a**) AF0; (**b**) AF1; (**c**) AF2; (**d**) AF3).

The microscopic morphology on the fibers surface was examined with AFM. These images revealed the changes in surface structure of the specimens due to scCO_2_ processing clearly. The details of the roughness of different samples were examined and shown in Table 5. It can be observed intuitively that the surface of AF0 was relatively flat, and the roughness was a commensurate lower value. After treatment with scCO_2_ Figure 12b, there were obvious protrusions and grooves, and the roughness value increased significantly. However, after modifying in scCO_2_ with glycidyl-POSS Figure 12c,d, the surface morphology has changed greatly. The corresponding R_a_ and R_q_ increased to 136.54 and 112.41 nm respectively. In particular, 2E4MZ as a curing accelerator can effectively promote glycidyl-POSS self-assembly and graft on fiber surface.

### 3.6. Monofilament Tensile Strength

**Figure 13:**
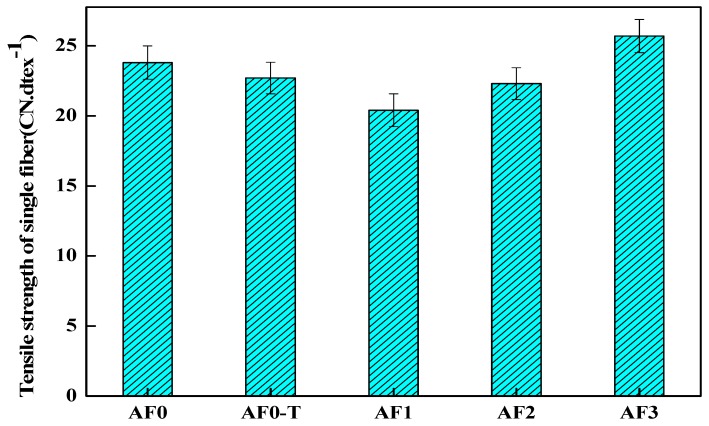
Tensile strength of aramid fiber before and after processing.

It can be seen in Figure 13 that the strength of AF1 decreased by 14.28% after scCO_2_ processing, which was directly related to the looser surface structure. The decrease in the tensile strength of the fiber after high temperature treatment is related to the change of its surface structure [3,22]. The scCO_2_ molecules which diffused into the surface of the fiber would improve the movement capacity of the fiber segments in some extent, and the segments would be adjusted, rearranged and even recrystallized. These molecular chains generated stress concentration under the action of stress, thus resulting in the decline of fiber filament strength [11,12]. Nanosized glycidyl-POSS permeated into the surface of the fiber under the action of scCO_2_ (AF2), which can reduce the damage of scCO_2_ to the fiber. As a curing accelerator, 2E4MZ promoted the ring-opening reaction and the grafting (or self-assembly) of glycidyl-POSS on the fiber surface, which was favorable for the improvement of fiber mechanical properties (AF3). Compared with AF0, the tensile strength of AF3 increased 8% to 25.7 cN·dtex^−1^.

### 3.7. The Mechanical Performance

**Figure 14:**
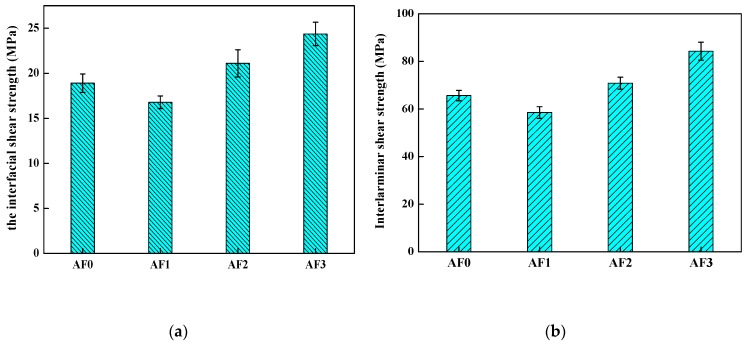
The mechanical performance of aramid fiber/epoxy with different processing. ((**a**) the interfacial shear strength; (**b**) the interlaminar shear strength).

To evaluate the influence of the grafted AF on the interlaminar shear strength (ILSS), the composites which were made of epoxy and AF treated with different type of processing were tested by inserting a grafted fiber layer in the AF/epoxy composites. The results of the micro-bond pull-out test and the measurement of the ILSS were both shown in Figure 14. As shown, the ILSS of AF1 reduced in comparison with AF0 (from 65.6 to 58.5 MPa), and the weakening of interlaminar shear strength well-correlated with the IFSS (from 18.90 to 16.79 MPa). While AF2 exhibited a higher ILSS (70.85 MPa) than that of AF0, ILSS of AF3 reached a new plateau of 84.26 MPa using 2E4MZ, as observed in Table 6. After scCO_2_ processing, the epoxy resin (epoxy micro-droplet) can easily penetrate the surface molecular chain of the fiber, the abundant carboxylic acids and amine functional groups could provide many reactive anchoring sites for the cross-linking of the epoxy resin, thus increasing adhesion performance. Moreover, a large number of glycidyl-POSS grafted (or self-assembly) on the surface of the fiber, improved the level of roughness after using 2E4MZ, and was critical for the adhesion. Although the value of IFSS in our research was not better than that of the processing of macromolecules grafting on the aramid fiber (increased by 34%) [1,3], the ILSS also increased significantly with the increase of IFSS. The increment of ILSS (25.33%) was much higher than the reports in the literature (14.7%) [23], which showed that the aramid fiber was modified according to our processing of aramid fiber-reinforced composites, which exhibited better properties.

**Figure 15:**
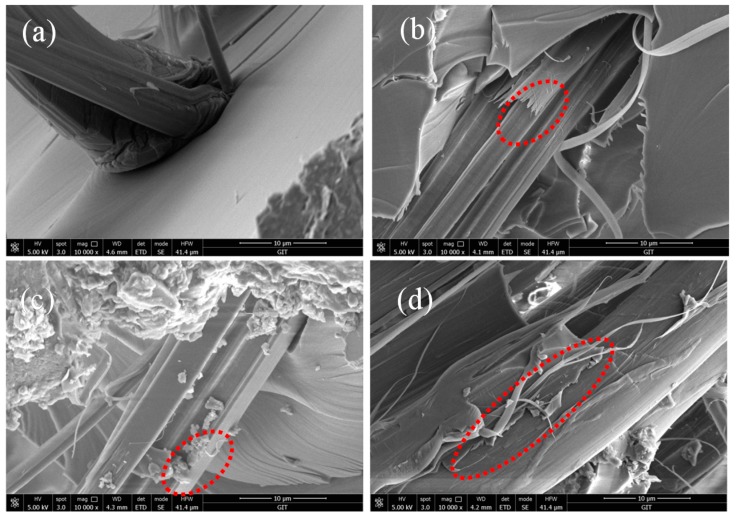
SEM images of AF/EP composites before and after modification. (**a**) AF0; (**b**) AF1; (**c**) AF2; (**d**) AF3.

To further evaluate the ILSS of fiber/epoxy composites, the impact fractured surfaces were examined under SEM. The fracture of AF0 is perfectly flat, as well as the fiber surface, and there was no adhesive epoxy resin on the fiber surface. Due to the action of scCO_2_, the roughness of AF1 increased and the surface structure was destroyed. The fiber was torn under an external force impact Figure 15b. This indicated that the decrease of ILSS was mainly caused by the decrease of single fiber strength, not adhesion [21,24,25,26,27]. After glycidyl-POSS processing, the polarity of AF2 increased and more epoxy resin was adhered to the fiber surface Figure 15c. On the fractured surface of AF3 Figure 15d, many epoxy debris still adhered, which confirms the strong interfacial adhesion between AF3 and the epoxy matrix.

## 4. Conclusions

In this study, a new and simple solution dipping method, using glycidyl-POSS as a modifier to modify AF, and the AF/EP micro-bond pull-out test, were evaluated. The results showed that scCO_2_ processing can destroy the surface structure of the fiber to a certain extent, which is beneficial to the permeation and grafting of glycidyl-POSS. This method can effectively improve the roughness and polarity of AF. XPS showed the introduction of C–O and –COO–, which confirmed the existence of chemical reactions between groups. 2E4MZ not only promoted the grafting reaction of glycidyl-POSS but also intensified the self-assembly, both of which increased the roughness of the fiber. The ILSS of the modified AF/EP composites increased 25.33% to reach at 84.26 MPa. These results indicated that AF treated in scCO_2_ with glycidyl-POSS and 2E4MZ, which is a suitable way of fiber modification, can significantly improve the surface cohesiveness of AF reinforced composites.

## Figures and Tables

**Table 1:** Sample number and treatment method.

Samples	Treatment Method
AF0	Untreated
AF0-T	AF0 sample were treated under 150 °C for 1 h.
AF1	Only scCO_2_ processing then washed and dried.
AF2	Soaked in glycidyl-POSS solution at 25 °C for 10 min and then treated in scCO_2_, left to treat in an oven at 150 °C for 1 h, then washed and dried.
AF3	soaked in glycidyl-POSS solution (+5% 2E4MZ) at 25 °C for 10 min and then treated in scCO_2_, left to treated in an oven at 150 °C for 1 h, then washed and dried.

**Table 2:** Relative chemical composition and atomic ratios determined by XPS for AF before and after processing.

Sample	Chemical Composition (%)			Atomic Ratio		
	C 1s	O 1s	N 1s	Si	C/N	O/N
AF0 AF0-T	77.16 83.40	12.12 9.51	10.72 7.09	0 0	6.36 11.7	1.13 1.34
AF1	83.50	9.01	7.49	0	11.14	1.20
AF2	72.49	15.98	6.28	5.25	11.54	2.54
AF3	66.13	17.89	2.94	13.04	22.49	6.08

**Table 3:** Results of deconvolution of C 1s for aramid fiber before and after processing.

Sample	Relative Area of Different Chemical Bonds (%)			
	C–C and C=C	O=C–N–H(C=O, C–N)	C–O	–COO–
AF0 AF0-T	88.15 81.55	11.85 18.45	0 0	0 0
AF1	82.54	17.46	0	0
AF2	79.93	16.84	2.49	0.74
AF3	68.32	8.86	21.74	1.08

**Table 4:** Comparison of crystalinity structure parameters of aramid fiber before and after treatment.

Sample	2θ (°)		D (nm)		FWHM (°)		Xs (nm)	Xc (%)
	[110]	[200]	[110]	[200]	[110]	[200]		
AF0	20.72	23.03	4.27	3.85	1.629	1.699	4.8	82.16
AF1	20.34	22.63	4.36	3.92	1.439	1.589	5.4	77.10
AF2	20.30	22.60	4.35	3.91	1.428	1.563	5.6	80.05
AF3	20.15	22.35	4.41	4.03	1.419	1.512	6.1	74.54

Xc—crystallinity; d (nm)—interplanar spacing; Xs—average crystallite size.

**Table 5:** Surface roughness of aramid fiber before and after processing.

Sample	AF0	AF1	AF2	AF3
Rq/nm	6.77	32.26	54.55	136.54
Ra/nm	4.97	29.49	41.47	112.41

**Table 6:** The interfacial shear strength (IFSS)/interlaminar shear strength (ILSS) value of different specimen.

Micro-Droplet Sample	IFSS (MPa)	ILSS (MPa)	Increase (Compared with AF0 IFSS/ILSS)
AF0	18.90 ± 1.02	65.63 ± 2.14	0/0
AF1	16.79 ± 0.70	58.51 ± 2.46	−11.16%/−10.84%
AF2	21.10 ± 1.50	70.85 ± 2.51	11.64%/7.95%
AF3	24.37 ± 1.29	84.26 ± 3.78	28.94%/25.33%
